# Pharmaceutical Chaperones and Proteostasis Regulators in the Therapy of Lysosomal Storage Disorders: Current Perspective and Future Promises

**DOI:** 10.3389/fphar.2017.00448

**Published:** 2017-07-07

**Authors:** Fedah E. Mohamed, Lihadh Al-Gazali, Fatma Al-Jasmi, Bassam R. Ali

**Affiliations:** ^1^Department of Pathology, College of Medicine and Health Sciences, United Arab Emirates UniversityAl Ain, United Arab Emirates; ^2^Department of Pediatrics, College of Medicine and Health Sciences, United Arab Emirates UniversityAl Ain, United Arab Emirates; ^3^Zayed Bin Sultan Center for Health Sciences, United Arab Emirates UniversityAl-Ain, United Arab Emirates

**Keywords:** lysosomal storage disorders, pharmaceutical chaperones, proteostasis regulators, missense mutations, conformational disorders

## Abstract

Different approaches have been utilized or proposed for the treatment of lysosomal storage disorders (LSDs) including enzyme replacement and hematopoietic stem cell transplant therapies, both aiming to compensate for the enzymatic loss of the underlying mutated lysosomal enzymes. However, these approaches have their own limitations and therefore the vast majority of LSDs are either still untreatable or their treatments are inadequate. Missense mutations affecting enzyme stability, folding and cellular trafficking are common in LSDs resulting often in low protein half-life, premature degradation, aggregation and retention of the mutant proteins in the endoplasmic reticulum. Small molecular weight compounds such as pharmaceutical chaperones (PCs) and proteostasis regulators have been in recent years to be promising approaches for overcoming some of these protein processing defects. These compounds are thought to enhance lysosomal enzyme activity by specific binding to the mutated enzyme or by manipulating components of the proteostasis pathways promoting protein stability, folding and trafficking and thus enhancing and restoring some of the enzymatic activity of the mutated protein in lysosomes. Multiple compounds have already been approved for clinical use to treat multiple LSDs like migalastat in the treatment of Fabry disease and others are currently under research or in clinical trials such as Ambroxol hydrochloride and Pyrimethamine. In this review, we are presenting a general overview of LSDs, their molecular and cellular bases, and focusing on recent advances on targeting and manipulation proteostasis, including the use of PCs and proteostasis regulators, as therapeutic targets for some LSDs. In addition, we present the successes, limitations and future perspectives in this field.

## Introduction

Lysosomal storage disorders (LSDs) are a heterogenic subgroup of more than 60 rare inborn inherited metabolic disorders (Burton, 1998; Winchester et al., 2000). LSDs were first diagnosed in the 19th century long before lysosomes were even identified in the cell by Christian de Duve in 1955 and therefore were not yet classified as LSDs at that time (de Duve et al., 1955; de Duve, 2005). The LSDs classification evolved subsequent to our improved understanding of the function of lysosomes and the identification of their biogenesis and enzymatic proteins. Lysosomes are specialized sacs housing hydrolytic enzymes to digest various cellular substrates to be recycled and delivered to targeted sites within the cell (Hunziker and Geuze, 1996). Individual LSDs are usually caused by deficiencies in specific lysosomal enzymes due to genetic defects in their coding genes (Grayson, 2016). Genetic defects will either disrupt the expression of the mutated protein or result in the expression of a structurally or functionally defective enzyme. In all cases, the residual enzyme activity reaches a level below the cellular threshold required for normal biological function. The major outcome of low lysosomal enzyme activity is the accumulation of its substrates in lysosomes leading to toxicity, cell swelling, and death (Ballabio and Gieselmann, 2009). This can occur in multiple tissues including muscle, eye, liver, spleen, bones, and joints. However, the most serious consequences arise when the deficient enzyme function is crucial for neuronal cells, which in fact is observed for most LSDs (Begley et al., 2008). A minor group of LSDs such as Mucolipidosis type II/III and Niemann–Pick disease type C1 are caused by defects in non-enzymatic lysosomal proteins, lysosomal membrane proteins and enzymatic co-factors rather than acidic hydrolases (Saftig and Klumperman, 2009).

In this review, we aim to present a general overview of the molecular and cellular bases of LSDs and highlight recent advances on our understanding of proteostasis manipulation as a therapeutic target for some LSDs. A brief description of the normal proteostasis and lysosomal network has been stated with a deeper insight into the biochemical and molecular mechanisms underlying different LSDs. Currently available LSDs treatments aim to reduce the accumulation of substrates in lysosomes. Enzyme replacement therapy (ERT) has been approved long ago for several LSDs but recently small molecular compounds manipulating proteostasis have been introduced in clinics and research laboratories to overcome limitations and inadequacies of the previous therapies. Pharmaceutical chaperones (PCs) are specific small molecular weight compounds that specifically bind and stabilize mutated enzymes while proteostasis regulatory compounds act generally on specific components of the proteostasis pathways to enhance protein folding by increasing cellular proteostasis capacity (Parenti et al., 2013). The focus of this article is to present recent advances on PCs and proteostasis regulatory compounds that were clinically and/or experimentally shown to be promising at correcting the defects and to summarize our understanding of their molecular bases by which they exert their effects.

## Lysosomal Storage Disorders: Genetics, Clinical Manifestations, and Epidemiology

Lysosomal storage disorders are mainly inherited as autosomal recessive disorders except for Fabry, Hunter, and Danon disorders which are inherited in an X-linked recessive manner (Ballabio and Gieselmann, 2009). The genotype and phenotype correlation of LSDs has been extensively studied to link symptoms to the genetic defect but little is known so far in understanding the cellular mechanisms by which the mutation causes the underlying disorder. Although disease symptoms are more linked to the type of the accumulated substrate and its location, however, LSDs pathogenic effects on lysosomal enzymes processes and pathways are not fully understood yet. All types of mutations were detected in LSDs with various effects resulting in heterogeneity of symptoms and severity. The most severe form of LSDs is the complete loss of a lysosomal enzyme due to protein truncations as seen with some indels (causing frameshifts) and non-sense mutations. In addition, for some LSDs, functional mRNA generation is blocked due to splice site mutations resulting in almost complete loss of the enzyme or very low residual activity when a small amount of the transcript is normally processed and translated (McInnes et al., 1992). Missense mutations are very frequent in LSDs but their effects are the most difficult to establish especially their cellular mechanisms. Missense mutations effects depend mostly on the site of change at the protein level. Amino acid substitution changes in the enzyme active site are believed to be the most deleterious leading to almost complete loss of residual enzyme activity (Zhang et al., 2000; Garman and Garboczi, 2004; Qian et al., 2015). Missense mutations occurring outside the active site may affect the folding properties and trafficking of the mutated protein, hence, its possible retention in the endoplasmic reticulum (ER) by the ER quality control machinery. ER retention leads to the complete loss of enzyme activity due to mislocalization of the lysosomal enzyme or premature degradation through the proteasomal degradation by endoplasmic-reticulum-associated protein degradation (ERAD) (Ron and Horowitz, 2005; Wang et al., 2011b). In both cases, the mutated enzyme does not successfully reach lysosomes to perform its function. In such cases, small molecular chaperones have been proposed and tested as potential therapies to correct the effect of such structural mutations (Khanna et al., 2014; Hossain et al., 2015; Laigre et al., 2016).

Lysosomal storage disorders are clinically heterogeneous with wide range of symptoms even within the same disorder. The main hallmark among all LSDs is the accumulation of metabolic substrates within lysosomes leading to cell dysfunction and eventually cell death. Accumulation of undigested metabolites results in the activation of several cellular pathogenesis pathways leading to multi-systematic clinical manifestations (Futerman and van Meer, 2004). LSDs are progressive with most of the patients born healthy with no signs of the disease. Symptoms start appearing within a period of time depending on the underlying mutation, affected tissue and the biochemistry of the accumulated substrate(s) (Platt et al., 2012). LSDs are classified based on the type of the accumulated macromolecules into glycan, lipid, and protein degradation defects in addition to subgroups with affected trafficking and lysosomal protein transporters (Murthy et al., 2010). Most of LSDs belong to the glycan defected subgroup harboring about 30 different disorders. Gangliosides, galactosylceramide, and sulfatide serve important functions in the brain tissue and patients with LSDs (as seen in GM1- and GM2-gangliosidosis) who show cellular accumulation of the mentioned substrates suffer from variant CNS complications ranging from seizures to intellectual disabilities (Sandhoff and Harzer, 2013). The severity of neurological presentation correlate with the site of accumulated substrates which can be the cortex, thalamus, cerebellum, and hippocampus (Platt et al., 2012). On the other hand, some of the LSDs do not have central nervous system (CNS) involvement. Recently, LSDs has been classified according to the defective protein as the previous classification can be misleading for some disorders (Wraith, 2011). For some LSDs more than one substrate is accumulated in tissues as seen in GM1-gangliosidoses patients who show defects in oligosaccharides, sphingolipids, and keratan sulfate degradation collectively (Sandhoff and Harzer, 2013). Mucolipidosis disorders on the other hand show glycoproteins and gangliosides aggregates rather than mucolipids as proposed by its name (Paik et al., 2005). Based on the disease age of onset, multiple LSDs like GM1-gangliosidosis are classified into infantile, juvenile, and adult forms where the classical LSD is the infantile form which is mostly associated with CNS manifestations leading to poor life expectancy of the affected child (Wraith, 2002). Symptoms might appear as early as in uterus or directly after delivery whereas in some cases the child is born healthy and symptoms appear after few months and progress, often rapidly. Adulthood form of LSDs usually have milder manifestation of the disease and patients tend to have better life expectancy (Platt et al., 2012).

In addition to the rarity of LSDs incidence, clinical phenotype overlapping between disorders as well as the delay or misdiagnoses of many disorders contribute to the low amount of data reported and the poor epidemiological coverage of LSDs. Individual LSDs are rare worldwide but collectively they are relatively common with a prevalence of ∼1/5175 live births (Sanderson et al., 2006). High frequency was found in an Emirati study conducted in 2013 with a total incidence of 27 per 100,000 live births which was close to the data obtained from a Portuguese study in 2004 (Pinto et al., 2004; Al-Jasmi et al., 2013). Gaucher is the most common type of LSDs worldwide with a ∼1/75,000 live births followed by Fabry disease with a 1/100,000 live births incidence (Grabowski, 2005; Germain, 2010). LSDs incidence on the other hand is relatively high in genetically isolated communities such as the Ashkenazi Jews who showed a prevalence as high as 1 in 855 live births for Gaucher’s disease (Vallance and Ford, 2003).

## Proteostasis and Lysosomal Maintenance

To understand the molecular and cellular mechanisms behind LSDs, we need to understand the concept of proteostasis and lysosomal dynamics. Proteostasis is the combination of multiple regulatory integrated systems including protein synthesis, structural folding, post-translational modification, trafficking and degradation (Labbadia and Morimoto, 2015). Lysosomal enzymes are glycoproteins synthesized in the ER with a specific N-terminal signal sequence. Several glycosylation events occur in the ER to enhance protein folding with the help of several ER-resident enzymes and molecular chaperones. Protein glycosylation and loss of the N-terminal signal initiate enzyme folding and translocation to the Golgi. Once in the Golgi, most lysosomal enzymes undergo a phosphotransferase and diesterase enzymatic processes to acquire a mannose 6-phosphate (M6-P) moiety that is important for protein translocation into lysosomes via M6-P receptors expressed on lysosomal membranes (Coutinho et al., 2012). The protein-receptor complex dissociates under lysosomal acidic environment where receptors are recycled back to Golgi for another round leaving the enzyme within lysosomes. Lysosomal acidity activates the trafficked enzyme through multiple proteolytic and/or folding processes to achieve the fully functional active enzyme conformation (Vellodi, 2005).

Proteins that fail to fold properly in ER, may aggregate disrupting cellular haemostasis in a condition known as ER stress (ERS). A cell under ERS activates various signaling pathways and processes including unfolded protein response (UPR) to restore its normal state through the expression of various genes functioning as chaperones to enhance protein folding, translational inhibitors to stop protein flux into ER, and activators of ERAD machinery (Xu et al., 2005). In the case of chronic UPR when a cell fails to reach haemostasis, specific apoptotic pathways are activated leading to cell death. UPR gene expression initiates the activation of three signaling pathways in the ER membrane; inositol-requiring protein 1 (IRE1), protein kinase R (PKR)-like endoplasmic reticulum kinase (PERK), and activating transcription factor 6 (ATF6) (Shen et al., 2004; Xu et al., 2005). The activation of IRE1 pathway by self-oligomerize and phosphorylation initiates the expression of the ERAD components. The PERK pathway on the other hand inhibits mRNA translation resulting in less protein flux to ER. ATF6 is an ER transmembrane transcription factor that is transported to Golgi where its cytosolic peptide gets cleaved by the site-2 protease (S2P) to be translocated to the nucleus and activates the transcription of ER protein folding chaperones (Tsai and Weissman, 2010; Walter and Ron, 2011).

As part of the UPR system, degradation of misfolded proteins that failed to fold properly is carried out by the ERAD machinery. ERAD is a highly orchestrated protein machinery that function in the cell’s ER and cytosol in four main steps. The process starts with recognizing misfolded proteins via highly specific chaperones through motifs like hydrophobic patches, *N*-glycan moieties, and disulphide bonds. Then substrates are targeted to retrotranslocation and ubiquitination. Retrotranslocation is an ATP-dependant step at which targeted proteins are translocated to the cytosol via the Cdc48 protein complex. Once in the cytosol, translocated proteins get polyubiquitinated by E3 ubiquitin ligases marking them for proteasomal degradation. Polyubiquitinated substrates are recognized by the proteasomal degradation machinery for deubiquitylation and breakdown into peptide fragments (Ruggiano et al., 2014).

Lysosomes are double membranous cytoplasmic organelle containing a group of hydrolases that digest and breakdown macromolecules that are delivered from inside or outside the cell. Materials from outside the cell are delivered through endocytosis via clathrin coated endocytic vesicles budded from plasma membrane which will fuse with endocytic vesicles budded from the trans Golgi network (early endosomes) (Settembre et al., 2013). As a lysosomal precursor, endosomes will mature to late endosomes by lowering its pH to 5.5 preparing a suitable environment for hydrolases enzymes. Lysosomes are also involved in processing and degradation of the cell’s own macromolecules and metabolites through autophagy to maintain cellular haemostasis (Vellodi, 2005). Based on substrate uptake, autophagy is classified into three types (Singh and Cuervo, 2011). Macroautophagy occur with encapsulating denatured macromolecules or damaged organelle with membranous structures generating autophagosomes that fuses with lysosomes to initiate the digestion process. Macromolecules that follow this path are RNA, carbohydrates and polyubiquitinated-proteins, small organelle like mitochondria, and ER segments (Eskelinen and Saftig, 2009). Macroautophagy malfunctions is common in different types of LSDs like impairment fusion of autophagosome to lysosome in mucolipidosis (Fraldi et al., 2010). Lysosomes also engulf cytosolic material by pinocytosis in a process called microautophagy. It is a non-selective autophagic pathway by which lysosomes directly engulf cytoplasmic materials via membrane invagination (Li et al., 2012). Defects in such processes are not well understood yet but it has been associated with Pompe disease (PD; Takikita et al., 2009). Autophagy can be selective in internalizing its material through a receptor mediated process that only binds proteins with the KFERQ motif in chaperone mediated autophagy (CMA) (Dice et al., 1990). Mutations in lysosome-associated membrane protein 2 (LAMP-2A) and mucolipin-1 receptors causes Danon and mucolipidosis IV disorders, respectively, by affecting the CMA process and substrate uptake to lysosomes (Fidzianska et al., 2007; Venugopal et al., 2009). Cellular pathways are always linked and working together to reach haemostasis. The crosstalk between autophagy, ERS and UPR is well maintained in the cell. The UPR-PERK signaling pathway induces the activation of autophagy to get rid of aggregated proteins. Autophagy is also activated when ERAD machinery is overwhelmed or fails to efficiently degrade certain types of highly structured proteins (Senft and Ronai, 2015).

## Biochemical and Cellular Mechanisms Underlying LSDs

**FIGURE 1:**
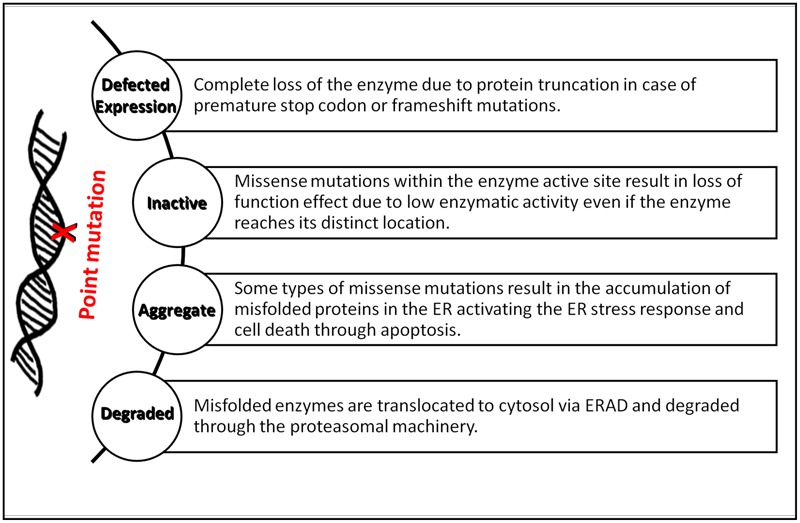
Summary of the major biological implications of point mutations in LSDs. The diagram summarizes the effects of point mutations on lysosomal enzymes’ synthesis, folding, and activity in forming LSDs.

Defects in any of the cellular or biochemical mechanisms that involves hydrolase enzyme syntheses, lysosomes biogenesis, lysosome-endosome system, or lysosome-autophagy system can lead to metabolites aggregation in lysosomes, hence, the occurrence of LSDs (Platt et al., 2012). Loss-of-function mutations in the genes encoding lysosomal enzymes may disrupt total protein synthesis or misfolding and retention in the ER. The latter defects are often caused by point mutations affecting protein maturation into its active conformation, trafficking to lysosomes and handled by ERAD (**Figure 1**). Most of ERAD substrates might be catalytically active but structurally unstable as seen in many disorders like Gaucher and Tay-Sachs disease (Schmitz et al., 2005; Dersh et al., 2016). UPR activation in some types of LSDs especially those involving CNS degeneration, as seen in GM1-gangliosidosis mice model for example, direct cells toward programmed cell death (Tessitore et al., 2004).

Several types of LSDs are associated with Ca^2+^ signaling impairment in different organelles. In Gaucher disease for example, ER calcium channel in neuronal cells are over activated due to metabolites accumulation resulting in high flux of calcium ions out of ER (Korkotian et al., 1999). Cytosolic Ca^2+^ is also elevated in Sandhoff, Niemann–Pick A and GM1-gangliosidosis disorders as the function of sarco/endoplasmic reticulum Ca^2+^-ATPase (SERCA) and inositol 1,4,5-trisphosphate-gated calcium channels are modulated, respectively, in these diseases (Pelled et al., 2003; Sano et al., 2009). On the other hand, alteration in mitochondrial Ca^2+^ haemostasis activates the apoptotic pathway in GM1-gangliosidosis and Mucolipidosis type IV diseases (Sano et al., 2009).

Accumulation of endolysosomes and autophagosomes have been noticed in some LSDs as a result of certain hydrolases deficiencies or substrate accumulation leading to the accumulation of more substrates and impairment of lysosomal pathways like the sphingolipid pathway in multiple LSDs (Prinetti et al., 2011). Alterations in the autophagy function contribute to the pathogenesis of many LSDs which leads to the accumulation of autophagosomes in affected cells and activation of cell death pathways. In GM1-gangliosidosis, neuronal ceroid lipofuscinosis, and Niemann–Pick type C disorders, overactivation of autophagy has been observed while in multiple sulfatase deficiency and mucopolysaccharidosis type IIIA (MPSIIIA) disorders autophagosome fails to fuse with lysosomes (Vitner et al., 2010).

## Diagnosis of LSDs

Diagnostic process of LSDs remains a challenge to many clinicians due to the clinical overlap of signs and symptoms between different disorders in combination with their rarity leading to misdiagnosis or significant delay of diagnosis (Kishnani et al., 2013). Most LSDs are usually presented with an early loss of acquired cognitive and motor skills in addition to various symptoms affecting different organs such as the spleen and liver (Wilcox, 2004). Patients’ may show neurological symptoms in combination with cardiac, musculoskeletal, and/or ophthalmologic features like corneal clouding (Staretz-Chacham et al., 2009). The correct diagnosis is a result of a productive collaboration between specialized clinicians and laboratory specialists. Generally, LSDs diagnosis is based on three major stages including preliminary clinical screening, biomedical testing, and genetic molecular testing (Filocamo and Morrone, 2011).

Patients are first assigned for clinical screening of the presented signs and symptoms. There are some common diagnostic features that have been clinically associated with specific disorders (Kingma et al., 2015). Features like cerebellar ataxia in GM1/2 gangliosidosis, Neuropathic pain and kidney failure in Fabry disease, Seizures and deafness in Krabbe, Hydrops fetalis in Farber, galactosialidosis, Gaucher, GM1 gangliosidosis and others, and ocular anomalies in Farber, galactosialidosis, GM1/2 gangliosidosis, sialidosis, and Gaucher (Kingma et al., 2015). Kingma et al. (2015) has suggested a diagnostic algorithm for a group of LSDs presented with dysmorphia, musculoskeletal manifestations and/or progressive cognitive impairment. Diagnosis is generally based on urine analyses of accumulated metabolites where glycosaminoglycans (GAGs) can be an indication of mucopolysaccharidoses (MPS) or multiple sulfatase deficiency. Sialic acid in urine is a common presentation of sialic acid storage disease while, oligosaccharides can be found in patients’ with GM1/2 gangliosidosis, fucosidosis, galactosialidosis, mannosidosis, and sialidosis. Diagnosis based on urine analysis carries a risk of false negative results in cases with mild loss of enzymatic activities as seen in MPS III or MPS IV. It is very important to know that LSDs are heterogenic with wide spectrum of signs and symptoms that may vary within the same disorder and therefore diagnosis based on clinical presentation only is often inconclusive and further analyses are required.

Measurement of residual enzymatic activities of different lysosomal enzymes are crucial in the diagnosis of primary LSDs. Enzymatic assays can be performed in various types of tissues expressing the targeted enzyme like serum, leukocytes, fibroblasts, and urine. Such assays are mostly fluorometric or colorimetric using artificial tagged substrates. The complete or excessive loss of enzymatic activity is enough to confirm the underlying diagnosis but in cases with normal enzymatic activities that are presented with clinical symptoms genetic testing is needed (Filocamo and Morrone, 2011).

Recently, genetic testing using whole-exome sequencing (WES) and whole-genome sequencing (WGS) have been successfully used in clinical diagnostics of many disorders including LSDs due to their fast, accurate and reduced costs (Katsanis and Katsanis, 2013). Analysis of DNA or RNA for mutations is required to identify LSDs with non-enzymatic lysosomal protein defects and it is used to confirm the results obtained from the enzymatic activity measurements (Platt et al., 2012). In post-mortem diagnoses, genetic testing is the best diagnostic approach because the only sample that can be collected is DNA. Results from genetic testing should be interpreted carefully as some genetic changes may just be polymorphisms as seen with c.1151G > A (p.S384N) variation in Maroteaux–Lamy syndrome (Zanetti et al., 2009).

## Therapeutic Strategies in the Treatment of LSDs: The Emergence of Proteostasis Manipulation as a Promising Target

Treatments for LSDs are mainly directed toward relieving the disease symptoms through supportive medical therapies. The first implemented therapy to correct the causative defect of the underlying disorder was in the 1990s (Parenti et al., 2013). Understanding the pathophysiology underlying LSDs have profound multiple therapeutic implications to increase the lost residual enzyme activities in different LSDs. In most LSDs, 10% of the residual enzyme activity is sufficient to enhance patients’ clinical presentation as well as only cells within the affected tissues need this enzyme enhancement for therapeutic benefits, unlike many other monogenic disorders that might require recovery in all tissues and organs. Based on LSDs pathophysiology, therapies are dedicated to either compensate for the enzyme loss or reduce the accumulated substrates.

As previously mentioned, hydrolases enzymes are transported to lysosomes via M6P receptors which are also expressed on cellular plasma membranes. A small portion of the ER synthesized enzymes are directly secreted extracellularly but are recaptured and internalized via the membranous M6P receptors to be delivered to lysosomes through the secretory pathway (Coutinho et al., 2012). Based on lysosomal cell biology, LSD deficient cells can take up exogenous enzyme through the M6P recapture mechanism. Therefore, therapies such as ERT, gene therapy, and stem cell transplant restore some of the lost enzyme using this principle.

### Enzyme Compensation Therapeutic Approaches

**FIGURE 2:**
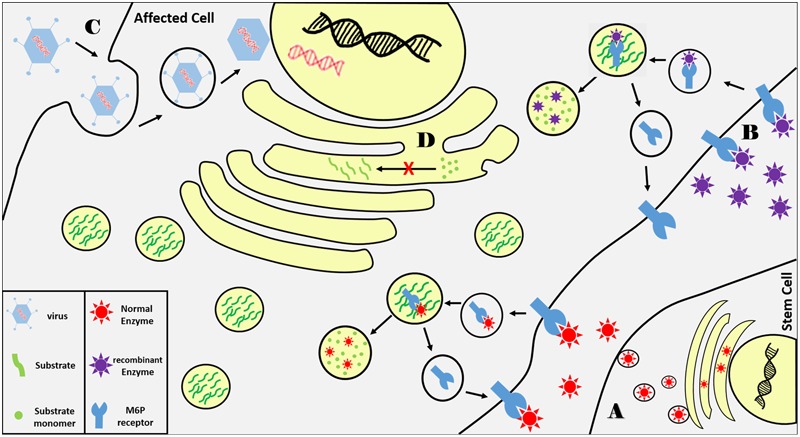
Summary of current LSDs therapeutic approaches. **(A)** Functional lysosomal enzymes are secreted from transplanted hematopoietic cells from matched donor as the enzyme is taken up via transmembrane M6P receptors on affected cells where they are trafficked to lysosomes. Normal enzyme breaks down accumulated substrates while captured receptors are recycled back to cell membrane. **(B)** Recombinant enzymes delivered via blood infusion are delivered to lysosomes via the same principle. **(C)** A wild type copy of the defected gene is delivered to affected cells via viral vectors. **(D)** Substrate synthesis is reduced using small molecular inhibitors that bind and inhibit enzymes involved in substrate biosynthesis.

Hematopoietic stem cell transplantation (HSCT) derived from matched bone marrow donors was the first therapeutic procedure used to treat LSDs (Malatack et al., 2003). Hematopoietic stem cells are delivered to patients from healthy matched donors to repopulate cells in the affected tissue and secrete functional lysosomal enzymes into the extracellular space and blood circulation where enzymes will be endocytosed by affected cells via M6P receptors (**Figure 2A**). Although this approach improves patients’ neurocognitive function, it is limited to few lysosomal disorders like mucopolysaccharidosis I and late-onset Krabbe disease. In addition, there are many safety concerns with HSCT and it is linked with high morbidity rate due to limited numbers of matched donors (Platt and Lachmann, 2009).

One of the most important therapeutic approaches in the treatment of LSDs is ERT (Rohrbach and Clarke, 2007). In ERT, patients receive periodic intravenous infusions of the missing lysosomal enzyme produced and purified by recombinant DNA technologies. The wild type enzyme is internalized by affected cells through M6P receptors into the endocytic pathway to compensate for enzymatic loss (**Figure 2B**). ERT was first implemented in Gaucher disease showing successful progression in thousands of patients and has been used to treat seven LSDs to date (Barton et al., 1990). It improves patients’ quality of life by ameliorating the hematological, biochemical, and visceral symptoms. In Fabry disease, ERT has positively affected cardiac, renal functions and reduced aggregated substrates in urine and plasma (Lidove et al., 2010). Similar effects have been shown in patients with PD who suffer from cardiomyopathy and skeletal muscle symptoms (Strothotte et al., 2010). The major limitation of ERT is the large size of the delivered recombinant enzymes which do not diffuse easily into all affected tissues such as bone, cartilage, and skeletal muscle. ERT also has low capacity to ameliorate neurological manifestations which are presented in two-thirds of LSD patients because the recombinant enzymes large and cannot cross the blood–brain barrier. There are numerous ongoing research efforts to overcome the delivery issue by modifying the recombinant enzymes with receptor moieties or chemically increase their half-lives.

Gene therapy is a promising therapeutic approach in treating many monogenic diseases. The principle of gene therapy is to deliver a wild type copy of the defective gene to the affected cells which in principle is feasible with monogenic disorders such as LSDs. Gene modification could be achieved using viral vectors *in vivo* by direct injection to affected tissue or *ex vivo* by manipulating patient’s hematopoietic stem cells (**Figure 2C**). Patients with LSDs are good candidates for gene therapy because, like ERT, correcting few cells might be sufficient to compensate for the enzyme loss based on the M6P reuptake mechanism as well as regaining only 10% of the residual activity might be significantly clinically beneficial. Unlike ERT, gene therapy is a one-time procedure that has a long-term effect and can be a suitable solution for those who suffer from very rare disorders that has no commercially available chemical therapeutics. Although gene therapy has promising future in treating LSDs, it has its own serious limitations and concerns. The major issue in gene modification via viral vectors is safety. Retrovirus and adenovirus vectors might cause cancer and may result in immune reactions toward the expressed enzyme. Gene therapy via intracerebral viral injection has been used to treat CNS-related symptoms in the mouse model of multiple sulfatase deficiency, however, this approach is still under intensive investigation (Spampanato et al., 2011).

The above mentioned therapeutic approaches are based on compensating for the lost enzyme in LSDs but another approach has been attempted aiming at reducing substrate synthesis and flux to lysosomes using small molecular inhibitors that bind and inhibit enzymes involved in substrate biosynthesis (**Figure 2D**). Substrate reduction therapy (SRT) has been approved for Gaucher and Niemann–Pick Type C diseases using Miglustat inhibitor which showed effective clinical improvements in both diseases (Giraldo et al., 2009). Recently, eliglustat (Cerdelga) has been approved for adults with Gaucher disease type 1 (Scott, 2015). More compounds are in preclinical and clinical trials for several LSDs such as MPS, Sandhoff disease, Fabry disease, and PD (Douillard-Guilloux et al., 2010; Marshall et al., 2010; Ashe et al., 2011). The main advantage in using substrate inhibitors is the ability of these compounds to reach different tissues including CNS due to their small molecular sizes but further studies are needed to accomplish the needed therapeutic goal.

### Correction of Proteostasis and Trafficking Defects as a Novel Approach

As already mentioned, defects disturbing any level of the normal proteostasis may result in a conformational disease at which the misfolded protein either aggregate forming toxic material as seen in many neurodegenerative diseases or most commonly loss of their biological function due to improper trafficking, ER retention, and/or degradation (Chaudhuri and Paul, 2006). Loss of function defects caused by missense mutations may affect protein folding, thermal stability, substrate binding, or enzyme turnover rate. Folding and maturation of proteins targeted to the secretory pathway is strictly monitored early within the pathway by a highly stringent ER quality control machinery (called ERAD) allowing only properly folded and assembled proteins to exit the ER to the Golgi complex for further post-translational modifications, targeting and trafficking to their final destinations including lysosomes (Ellgaard and Helenius, 2003; Chen et al., 2005). If a protein fails to reach its nascent conformation, due to a genetic defect for example, it will be recognized, retained in the ER and targeted for ERAD degradation. Thus, in many cases the underlying loss of function resulted from missense mutations might not be directly caused by catalytic activity loss and therefore rescuing the trafficking defect might lead to restoration of biological function. Many LSDs belong to the protein misfolding group of diseases especially those caused by missense mutations (Heine et al., 2007; Wang et al., 2011b). Lysosomal enzymes are acidic in nature and therefore have low thermal stability in the neutral environment of the ER. For some misfolded lysosomal enzymes, the corrupted 3D conformations lead to lower protein stability in ER and shorter half-lives.

#### Development of Low Molecular Weight Compounds as Protein Misfolding Correctors

**FIGURE 3:**
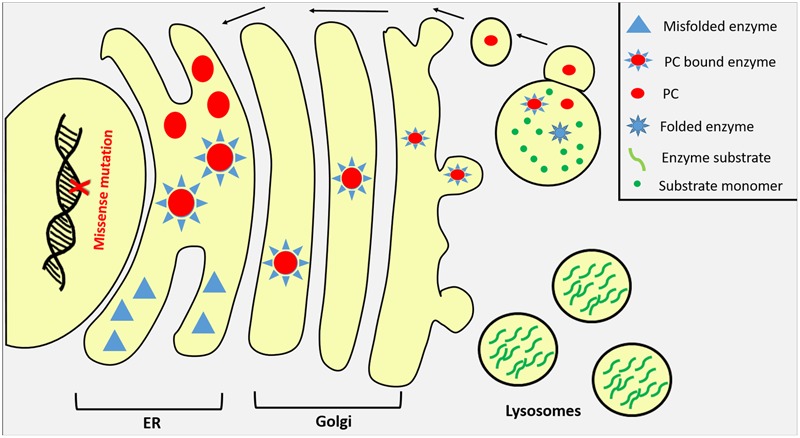
Pharmaceutical chaperones (PCs) as potential therapy for LSDs. Misfolded lysosomal enzymes due to missense mutations are usually retained in ER or prematurely degraded by the proteasomal apparatus in the cytosol. Once bound to its specific PC compound in the ER, misfolded enzyme undergoes proper folding and its stability increases promoting enzyme transportation to Golgi where it harbors its M6P residue directing the enzyme to lysosome through M6P receptors where substrates are accumulated. In lysosomes, PC compounds dissociate from lysosomal enzymes in response to the organelle acidic pH and substrate competition.

The extensive understanding of protein folding, proteostasis pathways, and lysosomal biogenesis have led to the development of several low molecular weight compounds that enhance protein folding and the rescue of some misfolded proteins from premature degradation (Lindquist and Kelly, 2011). There are three categories of small molecular weight compounds that have been described so far to restore the trafficking defects of ER misfolded proteins. The first are PCs that stabilize misfolded proteins by increasing their cellular levels as well as promoting their trafficking through the secretory pathway. PCs are usually inhibitory molecules that specifically and reversibly bind to target proteins to promote their conformational stabilization by promoting a more favorable free energy states compared to the unbound state at neutral pH (Valenzano et al., 2011). Once in lysosomes with its acidic environment and the availability of the enzyme’s natural substrates, the PC dissociates from the enzyme and thus restoring some of its catalytic activity (Wang et al., 2014). Enzyme ligands, agonists, and antagonists as well as cofactors and competitive inhibitors can act as PCs (**Figure 3**). The second class are chemical chaperones such as glycerol and DMSO but unlike PCs, these are unspecific molecules that alter the surrounding solvent conditions by sequestering water molecules increasing the unfolded protein free energy in a hydrophobic environment (Valenzano et al., 2011). The unspecific effect of chemical chaperones may result in cellular toxicity when premature protein intermediates are released from the ER (Selkoe, 2003). The third class of low molecular weight compounds are proteostasis regulators (PRs) that generally modulate the proteostasis network to increase its functional capacity. They work by enhancing the expression and functions of molecular chaperones and regulators of the ER quality control system to facilitate protein folding and minimize misfolding (Calamini and Morimoto, 2012). Proteostasis regulators manipulates the system through four possible mechanisms. Some PRs negatively affect protein production such as Guanabenz which has been used to inhibit global protein production in several diseases to decrease the load of molecular chaperone production resulting in less degradation of the targeted mutant protein (Tsaytler et al., 2011). Other regulators are used to either increase the production of molecular chaperones such as Geldanamycin or modify their function such as Carbamazepine which facilitates the toxic proteins clearance by enhancing autophagy via reducing the levels of inositol and IP_3_ (Sarkar and Rubinsztein, 2008; Nagai et al., 2010; Li et al., 2013). The fourth class of PRs function by directly manipulating the proteasomal system either by increasing its activity when cells are under stress or through inhibiting the system to prevent premature degradation (Twombly, 2003).

#### Pharmaceutical Chaperones as Therapeutic Agents in LSDs

Therapy using small molecular weight compounds in LSDs has been proposed due to the limitations of ERT and other conventional therapies. Unlike ERT, PCs have a broad tissue availability including the brain which recombinant enzymes cannot reach. In addition, the small molecular size of PCs allow easier membranous diffusion and thus delivering optimal concentration of the drug to affected cells (Beck, 2010). Patients under ERT receive lifelong intravenous infusion which requires hospital admission while PCs can be given orally. In addition, PCs safety profiles are expected to be superior to ERT because of the ability of the recombinant enzymes to elicit the patient’s immune system. Other treatments like SRT and hematopoietic stem cell transplant are restricted to few disorders while the gene therapy approach is still under research. Furthermore, small molecular compounds might be more suitable for correcting trafficking defects of proteins with missense mutations. As previously mentioned, missense mutations are common in LSDs and in most cases, they are located outside the enzyme active site and tend to affect protein folding, stability and trafficking (Muntau et al., 2014). PCs have the potential to correct the three-dimensional structure of mutated proteins and prevent their retention in ER or degradation as illustrate with examples presented in **Table 1**. For most LSDs, clinical manifestations develop when the residual enzyme activity falls below a threshold, which is in most cases around 10% of the wild type enzyme (Suzuki, 2014). Even restoration of 3–5% of the activity have been shown to slow down the clinical progress of the disease for several LSDs (Valenzano et al., 2011; Suzuki, 2014). Restoration of activities to such levels by PCs and PRs is therefore feasible, at least in some cases.

**Table 1:** Overview of some PCs in the therapy of LSDs.

Compound	Mechanism of action	Disease	Phase	Reference
Migalastat	Competitive inhibitor that stabilizes mutated enzyme and restores trafficking	Fabry Disease	Clinically approved by the European Union (EU) under the brand name Galafold^TM^ Phase III trial registration number: NCT00925301	Hughes et al., 2017 Benjamin et al., 2017
Ambroxol hydrochloride	pH-dependent mixed-type inhibitor of GCase that stabilizes mutated enzyme	Gaucher Disease	Pilot study	Narita et al., 2016
Isofagomine tartrate	Iminosugar restores correct confirmation and stability of mutated GCase	Gaucher Disease	Failed clinical trial	Shayman and Larsen, 2014
*N*-octyl-β-valienamine	GCase competitive inhibitor promotes mutated enzyme trafficking	Gaucher Disease	Experimental studies in cultured cells	Lin et al., 2004
*N*-acetylcysteine	Allosteric chaperone that increases physical stability of recombinant GAA in ERT	Pompe Disease	*In vitro* studies in patients’ cell lines	Porto et al., 2012
*N*-(*n*-butyl)deoxynojirimycin	Competitive inhibitor of GAA	Pompe Disease	*In vitro* and *in vivo* studies in patients’ cell lines and PD mouse model	Porto et al., 2009
α-lobeline and 3′4′7-trihydroxyisoflavone	Allosteric chaperones	Krabbe Disease	*In vitro* studies in Cos 1 and patient cell lines	Berardi et al., 2014
Azasugar	Competitive inhibitor	Krabbe Disease	Structural and biochemical studies	Hill et al., 2015
*N*-ocytl-4-epi-b-valienamine	Competitive inhibitor	GM1 Gangliosidosis	*In vitro* and *in vivo* preclinical studies	Hossain et al., 2016
5N,6S-(*N*’-butyliminomethylidene)-6-thio-1-deoxygalactonojirimycin	Competitive inhibitor	GM1 Gangliosidosis	*In vitro* and *in vivo* preclinical studies	Takai et al., 2013
Pyrimethamine	Competitive inhibitor	GM2 Gangliosidosis	Phase II	Clarke et al., 2011

Pharmaceutical chaperones have been initially tested to reverse the effects of *GLA* mutations resulting in Fabry disease (MIM 301500), an X-linked LSD due to α-galactosidase A (α-Gal A) enzyme deficiency. Patients with Fabry disease are presented with generalized vasculopathy with symptoms varying from acroparesthesia, angiokeratoma to progressive involvement of brain, kidneys, and heart due to the accumulation of globotriaosylceramide in these organs. Several α-Gal A mutants showed very low residual activities as a result of their extensively decreased stability in the ER leading to impaired trafficking and high protein turnover (Ishii et al., 1993). Although the enzyme’s ligand galactose increased the enzyme stability and activity in patients’ fibroblasts by binding to the active site, it was found to be of limited clinical use (Okumiya et al., 1995; Frustaci et al., 2001). Consequently, migalastat hydrochloride (1-deoxygalactonojirimycin) has been introduced as a potential therapy for Fabry disease. It is an iminosugar PC that specifically binds and stabilizes α-Gal A and has been shown to increase its residual activity in several tissues (Fan and Ishii, 2003). The effect of migalastat hydrochloride has been confirmed in several studies on fibroblasts from patients as well as in animal models and in clinical trials. However, this compound is still under investigation in phase 3 clinical trials due to the statistical insignificance of its use compared to the control group (Ino et al., 2013). A report of a phase 3 clinical trial comparing the safety and efficacy of migalastat to ERT in Fabry disease patients reported that migalastat is generally safe, tolerated, and significantly decreased the left ventricular mass index (LVMi) in affected patients compared to those who were on ERT (Hughes et al., 2017). Cardiac disease is a major complication and a major cause of death in Fabry patients due to heart failure and myocardial infarction. These outcomes might indicate the wider tissue distribution of the migalastat and its ability to penetrate cardiac tissues. Quality of life was improved in patients treated with migalastat based on progression analyses related to the renal, cardiac and cerebrovascular systems. Laboratory tests, ECGs, vital signs and physical exams of treated patients didn’t show any clinically relevant side effects. The compound didn’t show any positive enhancement in patients with non-amenable mutations who were responsive to ERT. It is worth noting that a pharmacogenetic study has developed a good laboratory practice (GLP)-validated assay in HEK293 cells to identify the α-Gal A mutants amenable to migalastat treatment (Benjamin et al., 2017). Out of 600 disease causing mutations, 268 were amenable to the drug. Mutations which were not responsive to migalastat included large deletions, insertions, truncations, frameshift, and splice-site mutations that mainly resulted in gross structural defects or complete loss of enzyme expression.

In addition, multiple PCs have been evaluated for the treatment of Gaucher disease (MIM 230800), one of the most common LSDs with progressive manifestations involving both CNS and visceral organs. It is caused by mutations in the β-glucosidase (GCase) resulting in reduced enzyme activity and accumulation of glucosylceramide metabolite in affected tissues. ERT is the main therapeutic approach to treat Gaucher patients but neurological manifestations have not been controlled with this approach (Desnick and Schuchman, 2012). Ambroxol hydrochloride (ABX) is an FDA approved drug that has been tested in Gaucher patients with neurological symptoms (Narita et al., 2016). It is a commonly used expectorant but pharmacological screening revealed a pH-dependent, mixed-type inhibition of GCase. It is a Biopharmaceutics Classification System (BCS) class I orally administrated drug that has high solubility and permeability (Maegawa et al., 2009). It showed significant improvements with p.N370S, p.F213I, and p.N188S mutated β-glucosidase and elevated the enzyme activity in N370S and L444P transgenic mice (Maegawa et al., 2009; Sanders et al., 2013). A pilot study conducted in five patients with neuronopathic Gaucher disease proved the ability of ABX to significantly improve patients’ neurological symptoms (Narita et al., 2016). Under high dosage, ABX significantly increased GCase enzymatic activity in patients’ lymphocytes and decreased metabolite accumulation in their cerebrospinal fluid. Symptoms like myoclonus, seizures, and pupillary light reflex dysfunction were improved in all patients and two of them could walk again after the recovery of a gross motor malfunction. Other compounds have reached the clinical trial stages in Gaucher such as Isofagomine tartrate (IFG) and the Amicus Therapeutics compound AT3375 (Sun et al., 2012). IFG showed promising results in several *in vitro* and *in vivo* studies. It increased residual enzyme activity in various mutated forms and tissues including the brain (Sun et al., 2012; Sanders et al., 2013). In phase I and II clinical trials, GCase activity was increased in both normal individuals and Gaucher patients but the drug failed because only one patient out of 18 showed significant clinical enhancement (Shayman and Larsen, 2014). It is worth noting that despite its failure, IFG showed promising results as a therapeutic strategy for Parkinson’s disease by increasing GCase activity in synucleinopathy mouse model (Richter et al., 2014). The valienamine derivative; *N*-octyl-β-valienamine (NOV), is a competitive inhibitor of β-glucosidase that showed significant results in cultured cells but these results were not translated into animal or human studies due to lack of clinical data (Ogawa et al., 1996). In F213I/F213I and F213I/L444P Gaucher cell lines, NOV increased cellular enzyme amount, resorted localization, and caused a dose dependant elevation in residual enzyme activity (Lin et al., 2004). Further studies are needed to test whether NOV works as a PC compounds for other mutants and test its capability to reach affected tissues including the brain in animal studies.

Pompe disease (MIM 232300) is an inherited metabolic cardiomyopathy LSD resulting from the accumulation of glycogen in muscle tissues. The disease is caused by mutations leading to low residual α-glucosidase (GAA) activity. Treatment for PD up to date is by recombinant ERT which was not effective in some patients due to its failure to reach the optimum therapeutic concentrations in affected skeletal muscles. Therefore, alternative approaches through PCs was needed to solve that issue. Consequently, *N*-acetylcysteine (NAC) compound was introduced as an allosteric chaperone for the α-glucosidase enzyme instead of using enzyme inhibitors which showed promising results *in vitro* (Porto et al., 2012). NAC was found to enhance enzymatic activity by stabilizing recombinant GAA at non-acidic pH. Based on this outcome, NAC can improve the efficacy of ERT in PD. Similarly, the PC *N*-(*n*-butyl)deoxynojirimycin (NB-DNJ) showed enhancement in GAA activity in combination with ERT proposing the effectiveness of the combination therapy of these compounds (Porto et al., 2009). NB-DNJ improved GAA trafficking to lysosomes, enhanced enzyme processing, and elevated enzyme stability in both patients’ fibroblast cells and PD mouse model.

Krabbe disease (MIM 245200) is a degenerative LSD caused by mutations in the galactocerebrosidase (*GALC*) gene. Some mutations lead to loss of activity due to improper processing and trafficking of the enzyme. PCs like 3′4′7-trihydroxyisoflavone and α-lobeline are weak inhibitors of GALC that rescued several missense mutated GALC enzyme in Cos 1 and patients’ cells (Lee et al., 2010; Berardi et al., 2014). Both compounds were found to act on the mutated enzyme via allosteric binding. Unlike 3′4′7-trihydroxyisoflavone and α-lobeline, azasugar is an active site competitive inhibitor of GALC that has a wide rage biodistribution and low toxicity (Horne et al., 2011). Directly targeting the enzyme active site made this PC more specific or GALC compared other compounds. Although it is still under biochemical and structural analysis, azasugar is considered as a potential PC compound in the development for Krabbe disease (Hill et al., 2015).

Low β-D-galactosidase (β-Gal) residual activity results in the accumulation of GM1 ganglioside and keratan sulfate substrates in various tissues leading to toxicity and deterioration of cells. Defective β-Gal is caused by mutations in the *GLB1* gene leading to two distinct LSDs, GM1-gangliosidosis (MIM 230500) which is a progressive neurodegenerative disorder and Morquio B (MIM 253010) which is a rare bone disease without CNS involvement. Treating GM1-gangliosidosis with the conventional ERT is not effective with the neurological symptoms because recombinant enzymes cannot cross the blood–brain barrier. SRT on the other hand is not specific and has serious clinical side effects. Different studies have tested multiple PCs to enhance stability and trafficking of misfolded β-Gal that retained some enzymatic activity. Galactose, 1-deoxygalactonojirimycin (DGJ) and its derivatives have shown relatively significant enhancement *in vitro* activity with multiple mutants. However, these compounds interacted with other enzymes such as α-Gal. Consequently, more specific PCs for β-Gal have been developed including the valienamine derivative *N*-octyl-4-epi-β-valienamine (NOEV) that enhanced the enzymatic activities of 22 out of 94 missense mutants, enhanced the breakdown of substrates, and showed clinically significant arrest of neurological progression in murine model of the disease (Higaki et al., 2011; Suzuki et al., 2012; Hossain et al., 2016). In addition, 5N,6S-(*N*′-butyliminomethylidene)-6-thio-1-deoxygalactonojirimycin (6S-NBI-DGJ) is an iminosugar derivative that binds to the enzyme active site to increase its stability and showed significant enhancement of residual enzyme activity in 24 out of 88 β-Gal mutants and thus leading to amelioration of CNS symptoms in mice model (Takai et al., 2013).

GM2 gangliosidosis is a group of two related LSDs caused by beta-hexosaminidase (Hex) enzyme deficiency resulting in the accumulation of GM2 ganglioside substrates in neuronal cells (Karimzadeh et al., 2014). It is characterized by progressive neurological deterioration leading to motor, cerebral and spinocerebellar malfunctions (Mahuran, 1999). Hex enzyme is a dimer protein composed of α and β subunits that are encoded by *HEXA* and *HEXB* genes, respectively (Gowda et al., 2017). Mutations in *HEXA* lead to Tay-Sachs disease (TSD, OMIN 272800) while defects in *HEXB* result in Sandhoff disease (SD, OMIN 268800) (Karimzadeh et al., 2014). These two disorders are clinically indistinguishable. Pyrimethamine (PYR) is a PC that reached phase II clinical trials to treat such disorders. It is an FDA approved antimalarial drug that binds to the active site of dihydrofolate reductase (DHFR) enzyme (Deloron et al., 2010; Chiricozzi et al., 2014). Due to structural similarities between Hex and DHFR active sites, PYR binds to Hex active site acting as a competitive inhibitor (Bateman et al., 2011). From two phase II clinical studies, PYR showed promising results with some mutants in SD and TSD diseases but not with all patients affected by late-onset form the disease (Clarke et al., 2011; Osher et al., 2011). There are still some concerns to be elucidated like drug side effects and pharmacokinetics in neuronal cells (Chiricozzi et al., 2014).

It is also worth noting that PCs monotherapy in some types of LSDs didn’t show clinically relevant enhancement but when combined with ERT, it synergistically elevates the residual activities in different systems. PCs not only stabilize mutated enzymes but also the wild type form. Based on this principle, PCs stabilize short lived recombinant enzymes and promote their trafficking. Combined therapy has been demonstrated in Fabry and Pompe disorders that showed an increase in enzyme activity and reduction in substrate aggregation (Benjamin et al., 2012; Porto et al., 2012).

#### Proteostasis Regulators as Promising Therapeutic Agents in the Therapy of LSDs

The main principle of using PR compounds in LSDs relies on their ability to reprogram and manipulate different pathways involved in proteostasis to enhance protein folding and stability, slow down premature degradation of the mutated enzyme and increase its cellular levels, and promote enzyme processing and trafficking to lysosomes (Benjamin et al., 2012). To date, there are no clinically approved PRs for the treatment of LSDs but studies in this area showed several promising compounds that can open a new avenue to ameliorate the effect of different LSDs-causing missense mutations or improve the efficacy of PC therapy in some disorders. PRs are mainly focused to modulate the proteasomal degradation pathway, heat shock response (HSR), calcium homeostasis, ERAD pathway, and lysosomal proteostasis (Song et al., 2013).

Several classes of proteasomal inhibitors like bortezomib and MG132 were found to improve the enzymatic function in multiple mutant forms of lysosomal enzymes with short half-lives (Shimada et al., 2011, 2015; Macias-Vidal et al., 2014). By inhibiting premature degradation of mutant enzymes via proteasomal inhibition, protein folding capacity will be improved due to the induction of several molecular chaperones like Heat-shock protein 90 (Hsp90), Heat-shock protein (Hsp40), Heat-shock protein (Hsp70), and Binding immunoglobulin protein (Bip) (Mu et al., 2008b). Bortezomib is an FDA approved proteasomal inhibitor that is used in the treatment of multiple myeloma (Richardson et al., 2003). It enhanced residual activity in both PC-responsive and PC-unresponsive PD mutants (Shimada et al., 2015). Improved enzymatic activity was mainly due to the increase in enzymatic maturation and trafficking. Bortezomib also have been shown to increase the efficacy of ERT in Pompe disease by the induction of immune tolerance as patients treated with Myozyme showed high antibody titers against recombinant GAA (Banugaria et al., 2013). Bortezomib showed similar results with missense mutations causing Niemann–Pick disease type C (Macias-Vidal et al., 2014). Multiple mutants were trafficked correctly to lysosomes and increased their activity resulting in lower cholesterol accumulation in affected fibroblasts. MG132 is an efficient, reversible proteasomal inhibitor that improved the stability, processing, cellular trafficking, and activity of several missense mutated GAA in PD patients’ fibroblasts (Shimada et al., 2011). It inhibits the proteasomal function via arresting the 26s proteasomal core activity. MG132 was found to reduce the accumulation of ganglioside products in sialidosis disease patients’ fibroblasts. Sialidosis is a rare autosomal recessive LSD characterized by defected sialidase enzyme due to genetic defects in the *NEU1* gene (d’Azzo et al., 2015). MG132 mediated the rescue of different sialidase mutants and restored enzymatic activity and localization to lysosomes (O’Leary and Igdoura, 2012). MG132 in combination with celastrol have shown partial restoration of protein folding, trafficking and function in several LSDs (Mu et al., 2008b). Celastrol has significantly amplified the effect of MG132 on enzymatic function and localization in sialidosis affected cells (O’Leary and Igdoura, 2012).

Celastrol is a HSR activator that inhibits the function of nuclear factor-κB (NF-κB) (Sethi et al., 2007). It showed promising enhancement in Gaucher and Tay-Sachs patient-derived fibroblasts by activating both HSR and UPR (Mu et al., 2008a). It increased the severe neuropathic L444P-GCase residual activity with 1.8 folds as well as other GCase missense mutations. It also exhibits a synergetic rescue effect in combination with *N*-(*n*-nonyl)deoxynojirimycin which is a PC compound used in treating Gaucher disease. Celastrol also increased β-hexosaminidase A residual activity in G269S Tay-Sachs fibroblasts with 1.6 folds. Celastrol interferes with Hsp90 (ERAD activator) recognition of GCase mutants, therefore, prevents premature degradation and enhances GCase enzymatic activity (Yang et al., 2014). GCase stabilization by celastrol was obtained due to the transcriptional activation of different molecular chaperones such as Hsp70, DnaJ homolog subfamily B members 1, 9 (DNAJB1/9) and BAG family chaperone regulator 3 (BAG3).

Like celastrol, geldanamycin, vorinostat, and LB-205 are used as Hsp90 inhibitors to restore proteostasis in several LSDs (Ingemann and Kirkegaard, 2014). Geldanamycin is a Hsp90 inhibitor inducing the heat shock transcription factor 1 (HSF1) activation which is the main regulator of different heat shock proteins involved in the induction of HSR (Zou et al., 1998). On the other hand, vorinostat and LB-205 are histone deacetylase inhibitors that inhibit Hsp90 deacetylation (Yang et al., 2013). The underlying inhibition results in less recognition of the mutant proteins in both Gaucher and Niemann–Pick disease type C, and hence less degradation while increasing the expression of folding/refolding molecular chaperones (Pipalia et al., 2011; Yang et al., 2013).

Several studies suggest that manipulating calcium homeostasis through targeting HSR proteins restored enzymatic homeostasis in several LSDs as seen with Gaucher disease, mucopolysaccharidosis IIIA, and α-mannosidosis (Mu et al., 2008). Diltiazem and verapamil are FDA approved L-type Ca^2+^ channels inhibitors in treating hypertension (Tartaglione et al., 1982). These compounds have low side effects compared to other agents used in treating LSDs and diltiazem crosses the blood–brain barrier (Naito et al., 1986). Inhibiting such channels minimizes Ca^2+^ depletion in ER leading to the upregulation of many ER molecular chaperones involved in protein folding, especially Hsp40 and BiP. Lacidipine is a more efficient and selective L-type Ca^2+^ channel inhibitor that has better ability to diffuse into affected cells because of its hydrophobic nature (Wang et al., 2011a). It showed better enzymatic enhancement with L444P GCase mutant compared to the previously mentioned blockers and found to upregulate Bip expression in treated cells.

The role of ERAD has been well demonstrated in the pathogenesis of LSDs (Jeyakumar et al., 2002; Ron and Horowitz, 2005; Schmitz et al., 2005). Kifunensine (Kif) and eeyarestatin I (EerI) inhibit ERAD by interfering with recognition or retrotranslocation, respectively (Wang et al., 2011b). Both inhibitors partially restored folding and activity of different mutants in Gaucher and Tay-Sachs fibroblast cells. Although EerI-mediated inhibition exhibited more efficient restoration of enzymatic function, Kif-mediated inhibition caused lower induction of UPR and apoptosis.

A more specific PR modulation that only affects lysosomal proteostasis was identified by overexpressing the transcription factor EB (TFEB) (Song et al., 2013). TFEB is a master modulator of lysosomal biogenesis and proteostasis as it controls the expression of the Coordinated Lysosomal Expression and Regulation (CLEAR) network (Segatori, 2014). The activation of the CLEAR network via TFEB facilitate the cellular clearance through autophagy and exocytosis (Palmieri et al., 2011). TFEB activation is also involved in lysosomal enzymes expression, folding, trafficking, and activation. Genetic or chemical activation of TFEB mediated L444P GCas folding and enhanced its enzymatic activity in patients’ fibroblasts (Song et al., 2013). In addition, it was found to rescue several HexA mutants associated with Tay Sachs disease.

## PCs and PRs Limitations in the Treatment of LSDs

Pharmaceutical chaperone therapy is considered a promising therapeutic approach for LSDs with conformational defects. However, there are still several major challenges for this approach to be fully translated into clinics. For example, this approach is mainly suitable for mutations causing the loss of function due to misfolding or trafficking of the protein. It is not suitable for mutations affecting residues in the active site or mutations affecting protein expression (Suzuki, 2014). To date, PCs are known to have mutation specific effect as not all missense mutations are responsive for a single compound (Takai et al., 2013; Hossain et al., 2015). Another major challenge in developing PCs as therapeutics is their specificity, especially with active site competitive inhibitors. For example, due to the structural similarities between GALC and B-Gal active sites, obtaining specific PCs for these enzymes is challenging (Deane et al., 2011). Additionally, high dosages of competitive inhibitor PCs may have adverse effects on enzymatic activities and hence loss of the target enzyme function. A suggested solution is to give the patient on PC therapy breaks and avoid continuous treatment (Ringe and Petsko, 2009). To enhance chaperones specificity, non-active site inhibitors have been developed but allosteric PCs showed significant off-target effects in some cases. Adjusting PC concentrations can be a solution to overcome the off-target effects (Valenzano et al., 2011). It has been suggested that combination therapy of PCs with proteostasis regulatory compounds or ERT can be a suitable solution for this challenge (Pastores, 2010; Kirkegaard, 2013). Finally, PCs efficacy is mainly measured by increase enzymatic activity which can be misleading in some cases. Different assays must be developed to measure different parameters affecting PC therapy (Spratley and Deane, 2016). PRs on the other hand act generally on different proteostasis pathways and are not specific for lysosomal proteins. The major consequence of the non-specific modulation of these pathways is the induction of ERS and UPR which can affect cellular function or viability (Song et al., 2013).

## Conclusion and Future Perspectives

Undoubtedly, there is an urgent need to find medical solutions and therapies for the untreatable as well as unsatisfactorily managed LSDs. LSDs are very diverse group of disorders with broad range of symptoms, distinct defected enzymes, and accumulated metabolites. The extensive understanding of proteostasis, lysosomal pathways, and pathophysiology of LSDs will aid in the development of novel approaches in LSDs treatments including precision and personalized therapies. Although therapies using PCs and protein regulators are still in their early stages, it showed promising results in some LSDs as well as the combination use of these compounds with conventional therapies showed enhanced clinical outcomes. Therefore, extensive studies are required to fully elucidate and understand the biological and cellular mechanisms of the LSDs-causing mutations as well as expand the search for small molecules to correct some of the underlying cellular defects as novel therapies for these life-threatening conditions.

## Author Contributions

FM searched the literature, wrote the draft of the manuscript and prepared the figures. BA initiated the project, edited and supervised the overall progress of the manuscript. FA-J and LA-G revised the manuscript. All authors revised and approved the manuscript.

## Conflict of Interest Statement

The authors declare that the research was conducted in the absence of any commercial or financial relationships that could be construed as a potential conflict of interest.
